# Identifying sex-linked metabolomic biomarkers in fish gonads after bacterial infection

**DOI:** 10.1007/s11306-025-02356-7

**Published:** 2025-11-15

**Authors:** M. Caballero-Huertas, C. Ladisa, S. López-Chillarón, S. Joly, H. R. Habibi, L. Ribas

**Affiliations:** 1 CIRAD, UMR ISEM, 34398 Montpellier, France; 2 ISEM, Université de Montpellier, CNRS, IRD, CIRAD, 34090 Montpellier, France; 3 Department of Biological Sciences, University of Calgary, Calgary, AB Canada; 4 Institut de Ciències del Mar, Consejo Superior de Investigaciones Científicas (ICM-CSIC), 08003 Barcelona, Spain; 5 PhD Program in Genetics, Universitat Autonòma de Barcelona (UAB), 08193 Bellaterra, Spain

**Keywords:** Outbreaks, Immune, Metabolome, Transcriptome, Sexual dimorphism, Aquaculture

## Abstract

**Purpose:**

Fish aquaculture faces sustainable production challenges. Among them are the pathogenic outbreaks that can compromise the health of the stocks from various perspectives, including broodstock reproduction. This study focused on identifying the metabolite alterations produced after a bacterial infection by *Vibrio anguillarum* in the gonads of European seabass (*Dicentrarchus labrax*). Sex-related response to the infection challenge was studied using a metabolomics approach.

**Method:**

The metabolome of testes and ovaries of adult fish were extracted and analyzed after 48 h of bacterial exposure by ultra-high-performance liquid chromatography-mass spectrometer using negative-mode electrospray ionization (ESI) (UHPLC-MS, Vanquish Horizon UHPLC coupled to a Thermo Fisher Scientific Q-Exactive HF). To further decipher the molecular events, metabolomic and transcriptomic data were interconnected.

**Results:**

In total, 97 metabolites were identified. In the ovary, uric acid, O-phosphoethanolamine, allantoin, and acetoacetic acid were more represented. By contrast, nine metabolites were altered after the infection in testes, including uridine, N-acetylglucosamine-6-Phosphate, and Gamma-aminobutyric acid (GABA). The most abundant metabolic cascades triggered by infection in ovaries were related to glyoxylate and dicarboxylate metabolism, nitrogen metabolism, and purine metabolism, while in testes, we observed changes in glycerolipid metabolism, glycerophospholipid metabolism, and galactose metabolism.

**Conclusion:**

The present results demonstrate, for the first time in fish, that changes in metabolic pathways induced following infection are sex-dependent. The findings will help develop sex-specific immune therapies, identify resistant phenotypes, and improve aquaculture infection protocols.

**Supplementary Information:**

The online version contains supplementary material available at 10.1007/s11306-025-02356-7.

## Introduction

Metabolomics has emerged as a powerful biochemical research tool in all areas of life sciences, including biomedicine and agriculture (Dixon et al., 2006; Gonzalez-Covarrubias et al., 2022; Putri et al., 2013). Quantitative metabolomics enables the characterization of the metabolome within a biological system, thereby providing an accurate snapshot of biological processes and responses to environmental perturbations. The metabolome is the complete collection of metabolites, or small molecule chemicals, found in an organelle, cell, organ, biofluid, or organism (Wishart, 2019). Shifts in the metabolic profile can take place, in part, due to alterations in gene expression, which are either regulated by physiological processes or triggered by environmental factors. Essentially, metabolites are the end products of molecular processes within a cell, stemming from DNA replication, RNA transcription, and subsequent translation into proteins that influence metabolism and metabolite concentration (Dettmer et al., 2007).

In the medical field, metabolomics has been used to identify biomarkers in various diseases (Johnson et al., 2016), to facilitate diagnostics, prognostics, and patient stratification. For example, studies have demonstrated the utility of metabolomics in the early detection of breast cancer (McCartney et al., 2018; Subramani et al., 2022) and type 2 diabetes (Guasch-Ferré et al., 2016). In toxicology, metabolomics has emerged as a powerful tool for risk assessment and toxicity characterization of chemical substances (Bottalico et al., 2021; Jordan et al., 2012; Olesti et al., 2021). By offering insights into perturbed metabolic pathways, metabolomics can aid in understanding toxic mechanisms of action and predicting potential impacts on human and environmental health (Bedia, 2022). In the context of aquaculture, metabolomics has shown immense potential for improving fish health and welfare, as well as monitoring environmental quality (Alfaro & Young, 2018). Recent studies have employed metabolomics to investigate stress responses in farmed fish, identify biomarkers of water quality, and optimize feeding regimes, and pathogen-associated biomarkers (Lulijwa et al., 2022; Wang et al., 2024; Xiao et al., 2019). Thus, while much research is still needed to identify the best candidates for improving fish farming, metabolomics, alongside traditional diagnostic tools, has emerged as a promising field.

The global aquaculture industry has experienced rapid growth, driven by the increasing demand for fish and fish products as a source of high-quality protein for human consumption (FAO, 2020). Thus, the sustainability and profitability of aquaculture are often challenged by various biotic and abiotic factors, including bacterial, viral, and parasite infections resulting from intensive culture (Bondad-Reantaso et al., 2005; Maqsood et al., 2011). These infections can affect the health of the broodstock and cause mass mortalities, which would directly compromise the progeny due to poor health or indirectly through the vertical transmission of certain pathogens (e.g., *Flavobacterium psychrophilum* in salmonids, Long et al., 2014), which may lead to economic losses at production facilities. During gonadal development, the immune system interacts with pathways related to reproduction, such as the biosynthesis of testicular androgens in goldfish (*Carassius auratus*), which depends on pro-inflammatory cytokines (tumor necrosis factor-alpha, TNFα, and interleukin 1 beta, IL1β) (Lister & Van Der Kraak, 2002). In these terms, the understanding of the crosstalk between the immune and reproductive systems is of great interest (Caballero-Huertas et al., 2020, Caballero-Huertas et al., 2024). Gonads are immune-privileged sites as they need to protect meiotic germ cells from immune responses (Maddocks & Setchell, 1990). In addition, the relationship between both systems has been verified through the study of molecular actors involved in species displaying environmental sex determination in which the immune stimulation at the larval stage can alter gonadal differentiation into male or female (Moraleda-Prados et al., 2021; Pradhan et al., 2012).

The immune response to pathogens may vary depending on the sex of the host since sexual dimorphism entails differences in body size, shape, traits, and color, as well as disease state (Mori et al., 2017). In fish, sexual dimorphism involves morphological characteristics and molecular mechanisms underneath (Caballero-Huertas et al., 2020). A pioneer finding in European sea bass (*Dicentrarchus labrax*) shown that after bacterial infections, the molecular pathways in testes are more altered than that in females (van Gelderen et al., 2025). In this context, integrating sex in understanding immune responses will positively influence fish welfare, economic impacts, and policymaking, leading to more tailored and effective aquaculture management (Caballero-Huertas et al., 2024). Although sex is a factor that influences the immune response (Klein, 2000; Klein & Flanagan, 2016), there is a lack of studies that compare the metabolome between sexes after an infectious process in fish. By analyzing systemic changes in metabolic profiles in the gonads, after an immune challenge through bacterial infection, we investigated the underlying metabolic mechanisms of immunity in the reproductive tissue, analyzed the specific metabolic alterations that occur in each sex, and correlated these results with the transcriptome. Thus, the present study may help to untangle the impact of infection on production and identify biomarkers of early infection in both males and females.

## Material and methods

### Fish husbandry

European sea bass fry (3.9 g) purchased at a commercial hatchery (ES390950000637, Sonrionansa, Cantabria, Spain) in January 2019 were raised at the Institute of Agrifood Research and Technology (IRTA, Tarragona, Spain) until April 2022 to provide a broodstock of 32 months of age. A total of 104 fish, with an average weight of 663 ± 162 g, were tagged and their sex determined. During the rearing period, all fish were fed commercial feed (Skretting) according to standardized feeding tables. They were housed in tanks with capacities ranging from 2000 to 10000 L, connected to an IRTAmar® recirculating aquaculture system (RAS). The system operated either in flow-through or RAS mode until the start of the challenge experiment. The photoperiod followed natural daylight, and water temperature varied naturally between 10 and 22 °C. Daily measurements included water temperature and dissolved oxygen (6.4 ± 0.6 mg L − 1; OXI330, Crison Instruments), while pH (7.4 ± 0.2; pHmeter 507, Crison Instruments), salinity (36‰; MASTER-20 T, ATAGO Co. Ltd), ammonia (0.14 ± 0.1 mg NH4 + L − 1), and nitrite (0.2 ± 0.1 mg NO2 − L − 1) levels were monitored weekly using a HACH DR9000 Colorimeter (Hach, Spain). In February, six weeks before the challenge experiment, the fish were individually tagged for identification with passive integrated transponder (PIT) tags (TROVAN, Madrid, Spain) and their sex was re-confirmed.

### Experimental design and infection treatments

To perform the bacterial challenge experiment, 40 healthy fish (20 males and 20 females) were randomly selected from the stock (see van Gelderen et al., in press). On April 5th, the fish were transferred to IRTA’s biosafety challenge facility and housed in two cylindrical tanks (2000 L) connected to a recirculating aquaculture system (RAS) unit (IRTAmar®). The RAS system was equipped with real-time monitoring of oxygen and temperature, as well as mechanical filtration, biofiltration, and ultraviolet water disinfection. Outflow water underwent chlorination and ozone treatment before discharge. The photoperiod followed natural daylight, and water temperature and salinity were maintained at 14.1 ± 1.1 °C and 32.3 ± 0.4 ppt, respectively.

Before bacterial inoculation, the fish were anesthetized with 100 ppm tricaine methane-sulfonate (MS-222, Sigma-Aldrich, Madrid, Spain). Each fish was intraperitoneally (IP) injected with a sublethal dose (LD20) of *Vibrio anguillarum* (10^5^ CFU/fish). The dose was determined from previous studies. Control fish received an IP injection of phosphate-buffered saline (PBS). Fish were divided between two tanks: One containing infected fish and the other containing uninfected fish. A total of ten female fish (703.72 ± 68.21 g weight and 34.05 ± 1.07 cm length) and ten male fish (680.50 ± 34.17 and 33,85 ± 0.61 cm length). The wellbeing of the fish was monitored daily, and no mortalities were observed throughout the experiment. After 48 h, fish were euthanized with an overdose of MS-222 and subjected to biometric measurements (standard length and weight). Gonadal tissues were collected and flash-frozen in liquid nitrogen and stored at − 80 °C. All the fish ovaries reached a similar maturation stage independently of the treatmen Gonadal maturation stages were visually assessed following the criteria outlined by Carrillo et al. (2015).

### Metabolomic analysis

Metabolite extraction, analysis, and identification were performed with samples obtained from testes and ovaries for each investigated group (Control: N = 5 testes, N = 5 ovaries) and *Vibrio anguillarum* treatment (INF: 5 testes, N = 5 ovaries). Metabolites were extracted in a 50% water–methanol solution, and the ratio sample (mg): methanol (μl) was normalized 1:20 with 50% methanol as described by (Ladisa et al., 2021). Homogenization of samples was carried out with a bead-beating homogenizer (TissueLyser II, QIAGEN) followed by centrifugation for 20 min at 13500 rpm. The supernatant was collected and stored at − 80 °C until the analysis was performed. An Ultra-High Performance Liquid Chromatography (UHPLC) coupled with a Thermo Fisher Scientific Q-Exactive HF mass spectrometer was applied to analyze the metabolic extracts. High-resolution full-scan MS data were examined with MAVEN freeware, and metabolites were identified using a standard library of m/z and retention times from previous analysis (Clasquin et al., 2012).

Metabolite relative quantification was carried out through the evaluation of the intensity of the analyzed peaks. To measure peak intensity, the top area was applied as a metric, representing the average intensity of the top three points (Clasquin et al., 2012). Metabolomics data were acquired at the Calgary Metabolomics Research Facility (CMRF).

### Metabolomic data processing

To avoid possible noise and artifacts, log transformation and Pareto scaling were conducted on the metabolites data set before statistical analysis (Katajamaa & Orešič, 2007). The clustering between the control and infected groups was assessed by a principal component analysis (PCA) and a partial least squares discriminant analysis (PLS-DA). The quality of the PLS-DA model, as well as the R^2^ and Q^2^ parameters, was calculated. Variable Importance in Projection (VIP) score > 1 was applied as a selection method and threshold to identify the differentially released metabolites (DRMs) that explain the greatest variability between the control and the infected groups measured by the PLS-DA model (Broughton-Neiswanger et al., 2020; Cho et al., 2008). VIP > 1 was used for hierarchical clustering (Ward clustering, Euclidean distance), univariate analysis *t*-test, and pathway analysis making use of Metaboanalyst 5.0 (Giommi et al., 2022).

### Bioinformatic analysis

To assess the statistically significant differences among experimental groups, a significant threshold of *P-*value adjusted using false discovery rate (FDR) < 0.05 was used. The DRMs were further investigated by volcano plot combining data of fold change and *t*-tests, allowing the identification of variables (metabolites) that differ significantly between the two investigated groups. The significant metabolites highlighted in the volcano plot were those that have a twofold change (up or down) and at least, *P*-value < 0.1 (Giommi et al., 2022). Pathway analysis was done using MetPA to combine the results obtained from Quantitative Enrichment Analysis (QEA) and Topological Analysis using the *Danio rerio* KEGG pathway library as a reference (Xia & Wishart, 2010). Data were represented by the -Log of the *P-*value of the result of the Quantitative Enrichment Analysis, and the impact factor was calculated from the topological analysis (Ladisa et al., 2021). Metabolic pathways with *P-*value < 0.05 were considered significantly impacted. In this context, an impact value of zero “0” shows that the number of connections that a node (metabolite) has with other nodes is relatively low, suggesting that the matched metabolites for a specific pathway have a marginal or relatively isolated position in the pathway.

### Transcriptomic and metabolic correlation

To explore the correlation between metabolites (VIP > 1) and significantly differentially expressed genes (DEGs, adjusted *P*-value ≤ 0.05) the transcriptomic data from van Gelderen et al., 2025 was used after updating the annotation with the latest E. sea bass genome version 114 (https://www.ensembl.org/Dicentrarchus_labrax/Info/Annotation). The MetaboAnalyst 6.0 online platform was utilized for generating the gene-metabolite interaction networks. The networks were displayed in Cytoscape by using the Prefuse Force Directed Layout. To detect highly reliable connections between the DEGs and metabolites through shared functional processes, human orthologs of the DEGs were identified using g:Profiler and used in the functional gene–metabolite network analysis. Genes that lacked a human ortholog gene symbol and a KEGG Orthology (KO) ID, or had no known interactions in the STITCH database, were excluded from the network. In total, in ovaries, from the initial list of 101 DEGs in European sea bass, 67 human orthologs with KEGG Orthology (KO) identifiers were retained for the interaction analysis. In the case of the testes, from 2,624 DEGs of European sea bass gene IDs, a total of 1,684 were included in the final network analysis. To simplify the complexity of the testes network, a restricted adjusted *P*-value ≤ 0.001 was explored; from 431 total DEGs a total of 278 contained a KO and were included in the analysis.

### Ethical statement

Fish rearing and maintenance complied with the European regulations of animal welfare (ETS N8 123, 01/01/91 and 2010/63/EU). The experimental design and treatments of this study were developed and conducted following approved guidelines by the Bioethical Committee of the Generalitat de Catalunya (reference code 9977) and the Spanish National Research Council (CSIC) Ethics Committee (reference code 1166/2021). The suitability of the fish facilities at ICM for animal experimentation was verified by the Ministry of Agriculture and Fisheries (REGA number ES080190036532).

## Results

### Sexual dimorphism in the released metabolites

**Fig. 1:**
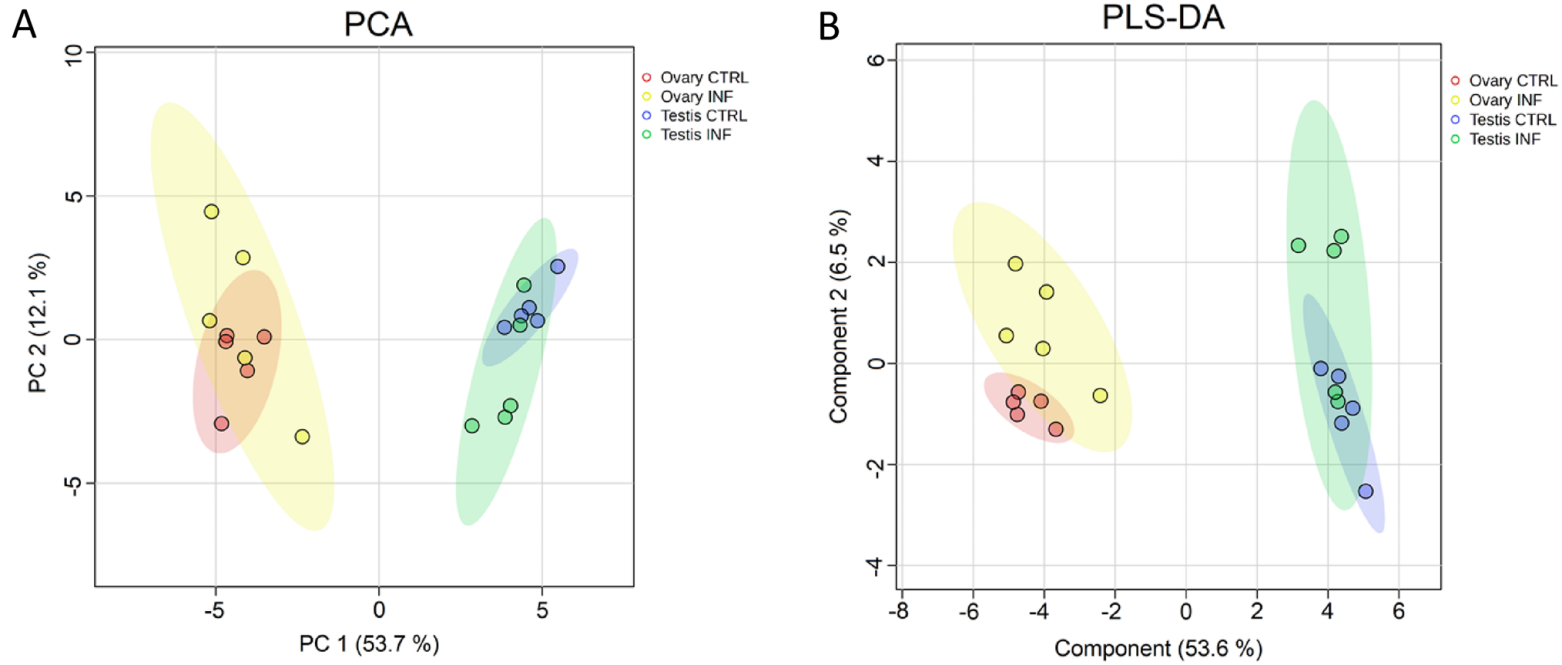
Principal Component Analyses, PCA (**A**) and partial least squares discriminant analysis, PLS-DA (**B**) analyses of control and infected in the gonads in European sea bass 48 h of bacterial post-injection

PCA and PLS-DA analyses of the four groups showed a strong separation between males and females. PCA and PLS-DA models represent the metabolic profile for both sexes and between control and infected fish for testes and ovaries (Fig. 1). In ovaries, the contribution rate of the supervised model (R^2^ = 0.9) and the predictability of the PLS-DA model (Q^2^ = 0.73) were more robust than in testes (R^2^ = 0.5; Q^2^ = 0.24). The PCA revealed 56.7% and 63.2% variance in ovaries and testes, respectively (Fig S1). Overall, the parameters confirmed that the differences between the control and infected groups for each tissue measured by the PLS-DA model are reliable and predictable.

**Fig. 2:**
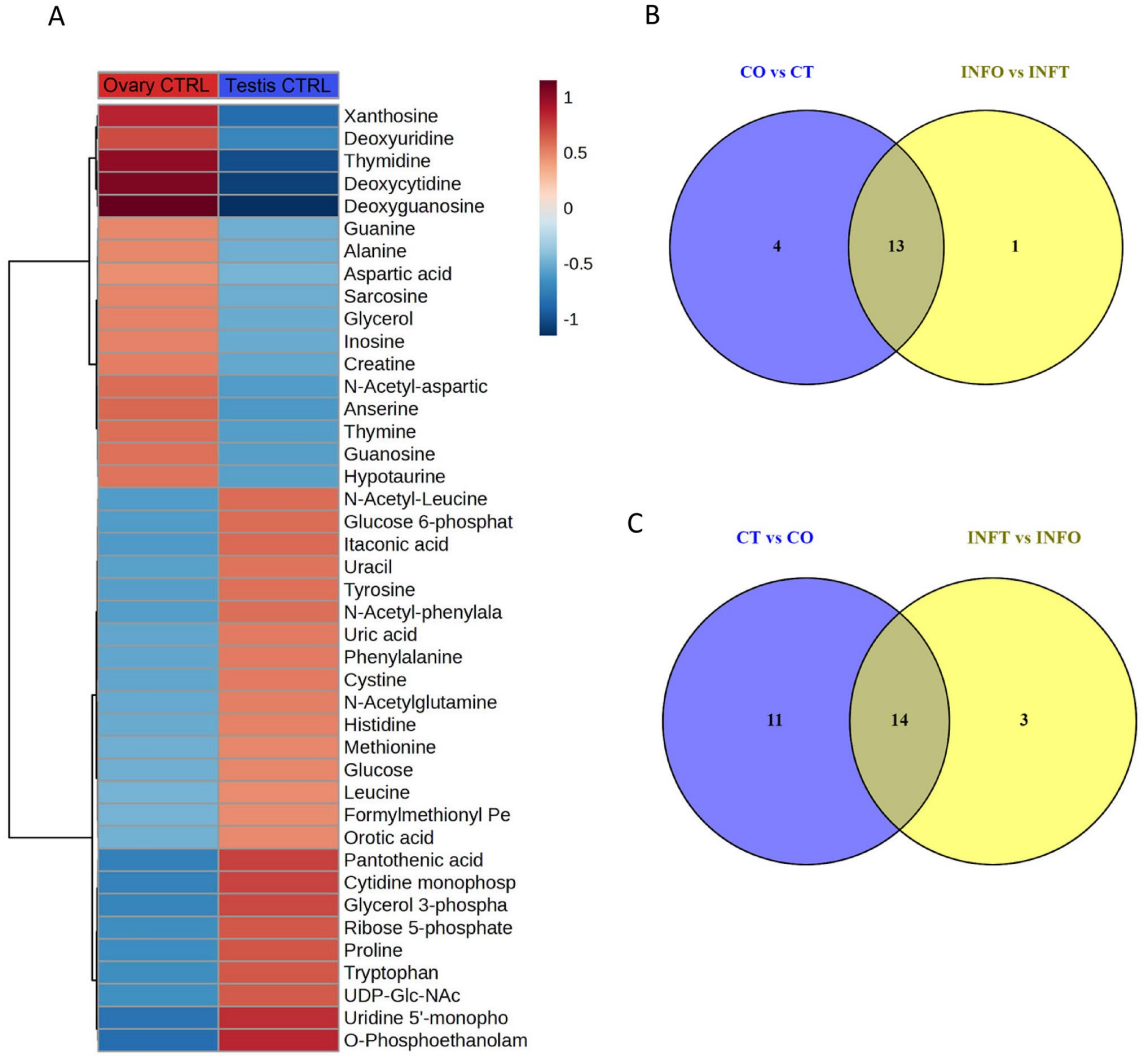
(**A**) Heatmap analysis and hierarchical clustering of the 42 sexually dimorphic metabolites in the gonads of European sea bass 48 h post-bacterial injection. (**B**) Venn diagrams of the more abundant metabolites in the ovaries compared to the testes in control and infected groups. (**C**) Venn diagrams of the more abundant metabolites in the testes compared to the ovaries in control and infected groups

**Fig. 3:**
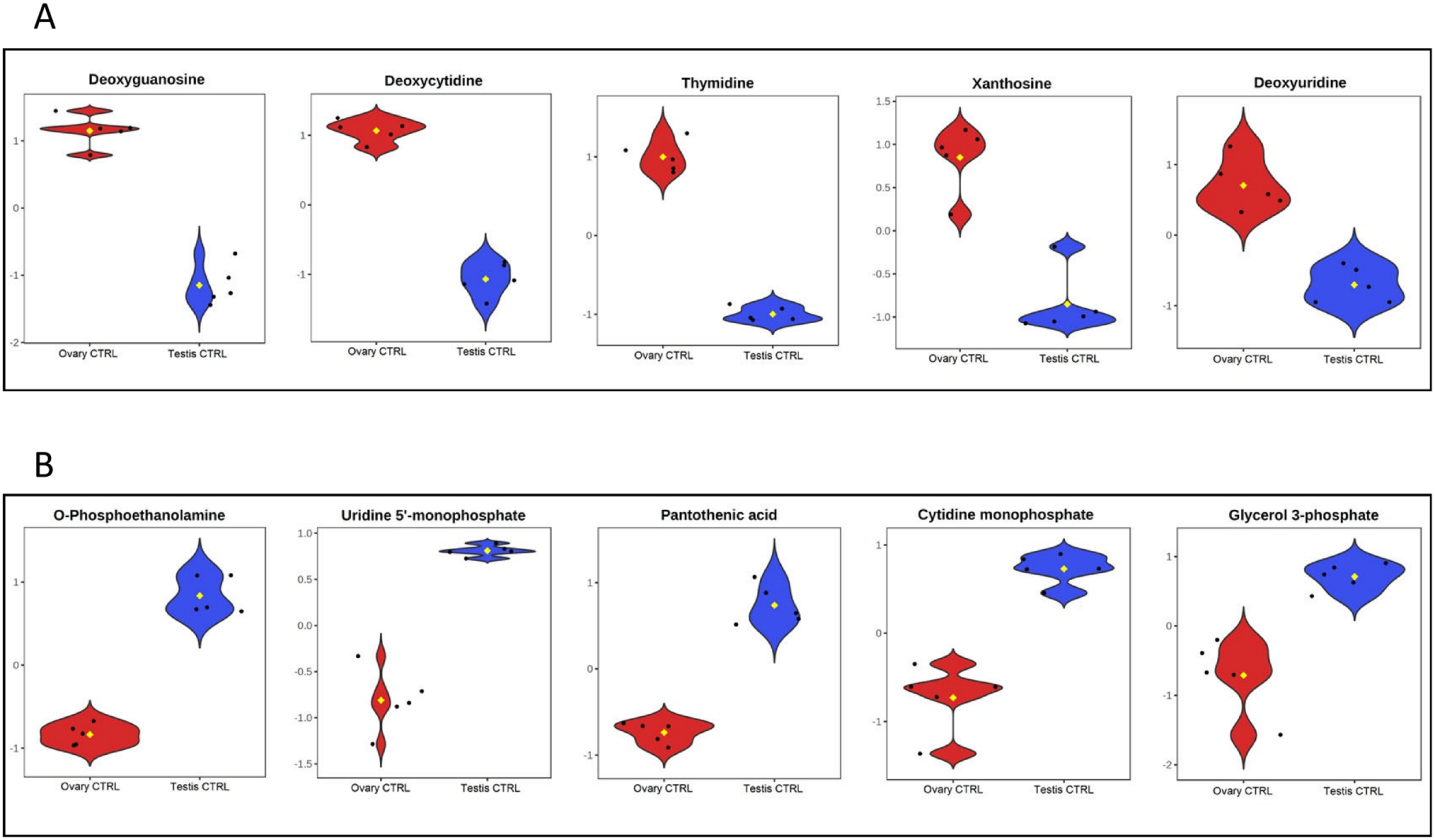
Violin plots showing the most significant (*P*-value < 0.05, twofold change) metabolites released in the ovaries (**A**) or in the testes (**B**) in European sea bass 48 h of bacterial post-injection

The comparison between ovaries and testes in control groups showed significant differences in 17 metabolites released in ovaries and 25 in testes, thus identifying 42 sexually dimorphic metabolites (Fig. 2A). The top 5 most abundant metabolites in control ovaries were deoxyguanosine (FC: 181.9, *P* = 8.9·E^−7^), deoxycytidine (FC: 169.1, *P* = 1.7·E^−7^), thymidine (FC: 158.1, *P* = 3.3·E^−8^), xanthosine (FC: 134.7, *P* = 0.1·E^−4^), and deoxyuridine (FC: 111.6, *P* = 0.1·E^−4^) (Fig. 3A). The top 5 most abundant metabolites in control testes were O-phosphoethanolamine (FC: 132.5, *P* = 4.9·E^−7^), uridine 5′-monophosphate (FC: 128.3, *P* = 6.3·E^−6^), pantothenic acid (FC: 116.9, *P* = 1.4·E^−6^), cytidine monophosphate (FC: 115.6, *P* = 5.0·E^−5^), and glycerol 3-phosphate (FC: 112.6, *P* = 4.5·E^−4^) (Fig. 3B).

**Fig. 4:**
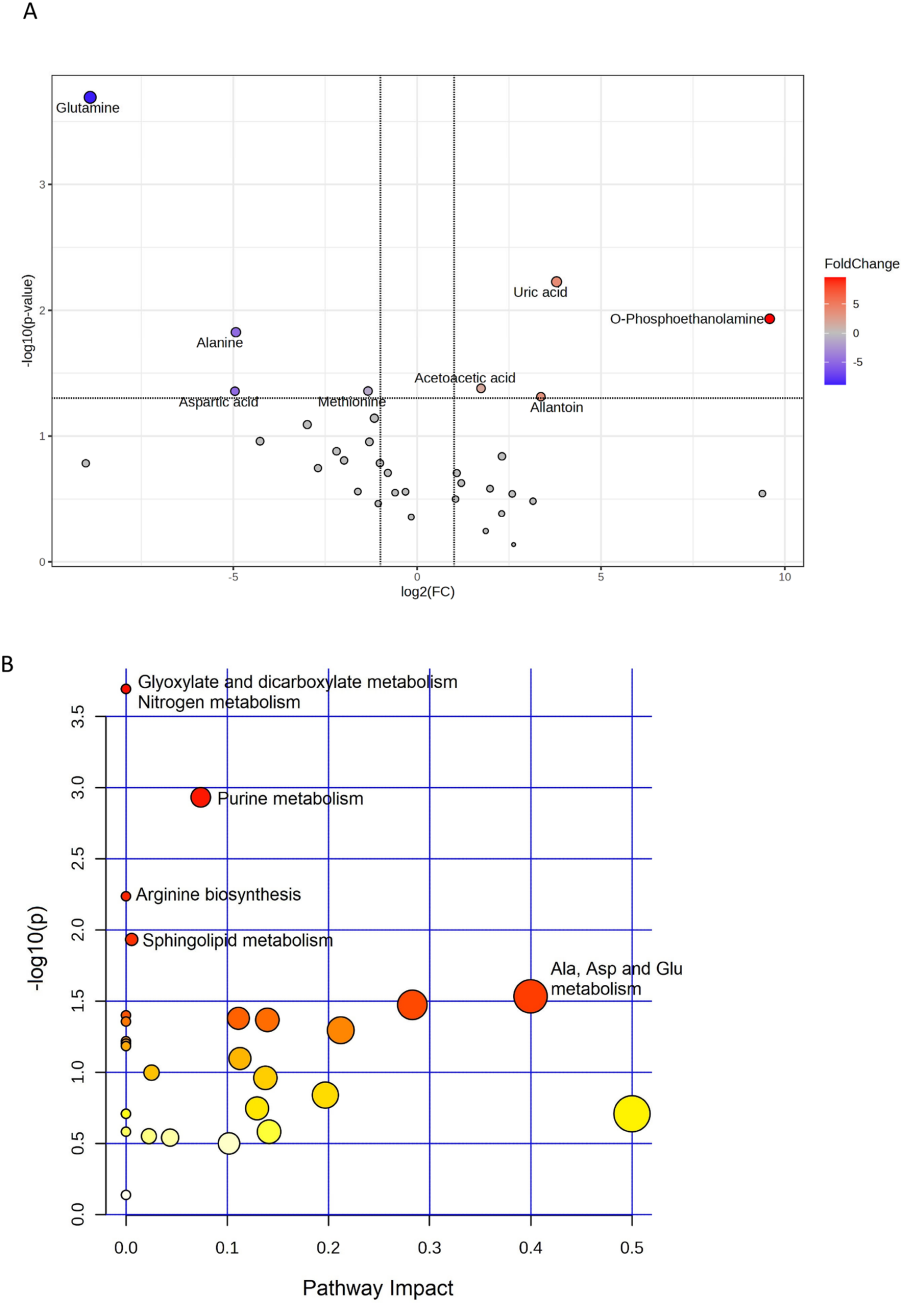
(**A**) Volcano plot of metabolites analysis found between control and infected groups after 48 h of bacterial post-injection in the ovaries. Colored dots represent the significantly different metabolites (*P*-value < 0.05, twofold change). (**B**) Pathway enrichment analysis in the ovaries after infections. *Danio rerio* Kegg pathway library was used as genome reference

**Fig. 5:**
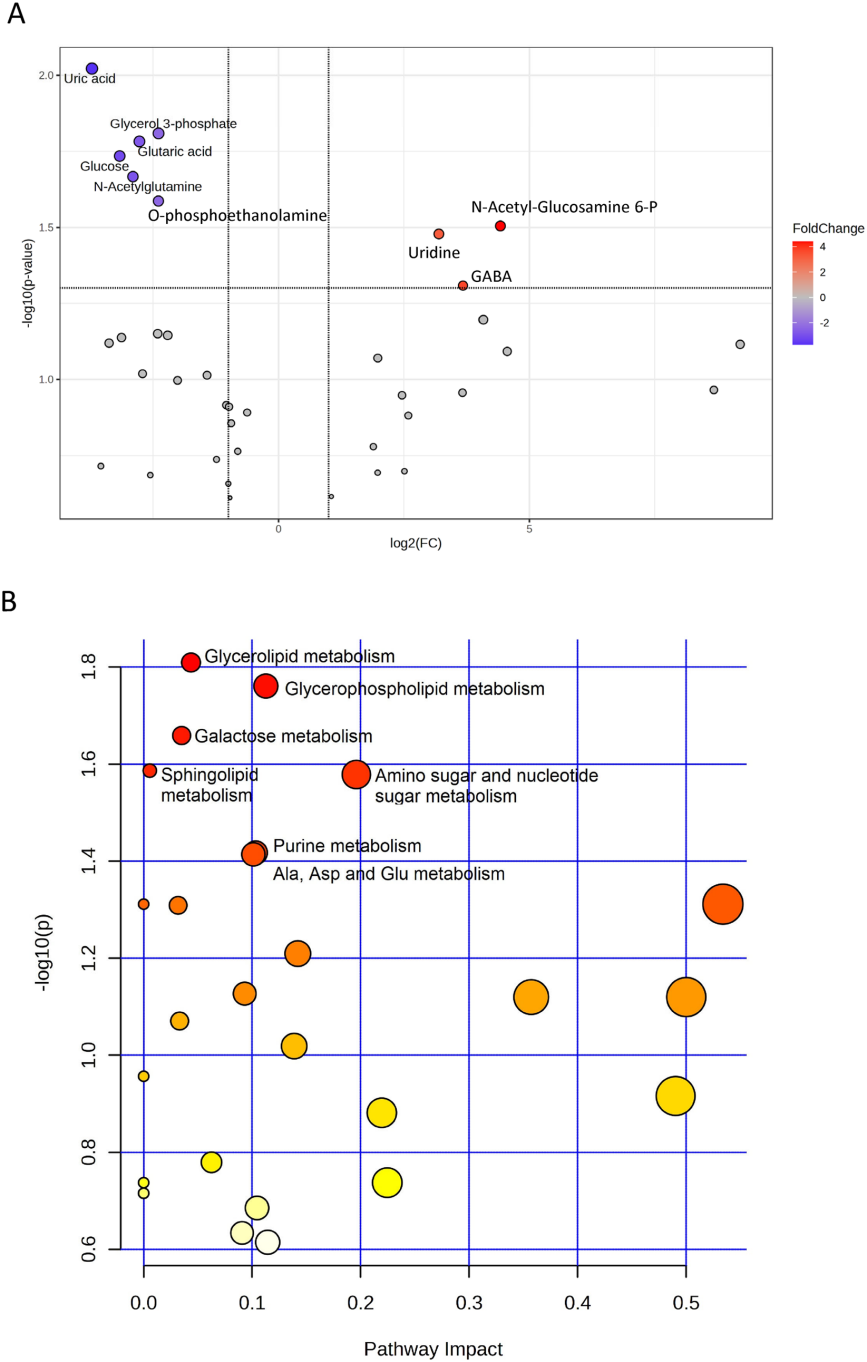
(**A**) Volcano plot of metabolites analysis found between control and infected groups after 48 h of bacterial post-injection in the testes. Colored dots represent the significantly different metabolites (*P*-value < 0.05, twofold change). (**B**) Pathway enrichment analysis in the testes after infections. *Danio rerio* Kegg pathway library was used as genome reference

There were 14 and 17 DRM in the ovaries and testes after the infections (Fig. 2B, C). In ovaries, 13 metabolites were released in both control and infected gonads while only one metabolite, glutaric acid, was differentially released due to the infection (Fig. 2B). In testes, 14 metabolites were released in both control and infected gonads, and three (cytidine, adenine, and valine) were more abundantly released due to the infection (Figs. 2C, 4, 5).

### Metabolites released in the ovaries after infection

**Fig. 6:**
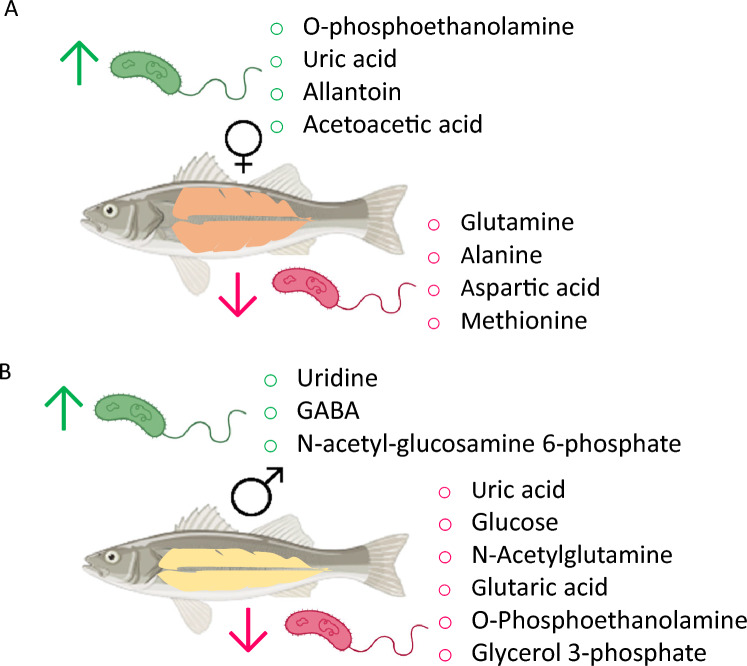
Summary of the metabolic alterations in the gonads (**A**, ovaries and **B**, testes) of European sea bass 48 h of bacterial post-injection. Green arrows show an increased release, while pink arrows show a decreased metabolite production after the infection

Using VIP analysis scores higher than 1,34 DRMs constituted the variability between infected and control groups in the ovary (Table 1). Among them, and considering the significance *P-*value < 0.05 and the twofold change of the volcano plot (Fig. 4A), eight metabolites were differentially released after the infection, among which four were more abundant (i.e., O-phosphoethanolamine, uric acid, allantoin, and acetoacetic acid) and four were less abundant (glutamine, alanine, aspartic acid, and methionine) (Fig. 6; Supplementary Fig S2). Among these metabolites, O-phosphoethanolamine (*P* = 0.01) was released 769 times more in the infected ovaries, while glutamine was reduced 473 times (*P* = 0.0002) (Table 2).

**Table 1:** Variable Importance in Projection (VIP) with a score > 1 analysis representing the differentially released metabolites (DRMs) in the European sea bass ovaries after 48 h post bacterial infection

Metabolite	VIP score
Glutamine	2.322
Uric acid	1.984
O-Phosphoethanolamine	1.823
Alanine	1.780
Acetoacetic acid	1.584
Methionine	1.578
Aspartic acid	1.554
Allantoin	1.547
Thymidine	1.524
Leucine	1.481
N-Acetylasparagine	1.346
Glutaric acid	1.329
Valine	1.323
Cystathionine	1.322
Threonine	1.255
Asparagine	1.250
Ribose 5-phosphate	1.236
N-Acetyl-aspartic acid	1.220
Uracil	1.201
Tyrosine	1.191
5-Hydroxy-tryptophan	1.112
Uridine 5′-monophosphate	1.099
Glucosamine 6-phosphate	1.099
Glycerol 3-phosphate	1.097
Deoxycytidine	1.073
4-Hydroxyproline	1.066
Guanosine	1.062
Glucose 6-phosphate	1.056
Glutamic acid	1.037
5-Hydroxylysine	1.033
N-Acetyl-phenylalanine	1.028
N-Acetylthreonine	1.026
Urocanic acid	1.024
Xanthosine	1.003

**Table 2:** Variable Importance in Projection (VIP) with a score > 1 analysis representing the differentially released metabolites (DRMs) in the European sea bass testes after 48 h post bacterial infection

Metabolite	VIP score
Uric acid	1.902
Glycerol 3-phosphate	1.820
Glutaric acid	1.809
Glucose	1.788
N-Acetylglutamine	1.758
O-Phosphoethanolamine	1.720
Uridine	1.679
N-Acetyl-Glucosamine 6-P	1.665
GABA	1.570
Glucosamine 6-phosphate	1.500
4-Hydroxybenzaldehyde	1.469
Aconitic acid	1.466
Suberic acid	1.461
Phenylalanine	1.448
Guanosine	1.445
Glutamic acid	1.429
Sorbitol	1.413
Tryptophan	1.375
Adenosine monophosphate	1.371
Proline	1.358
Cytidine	1.334
Ribose	1.327
N-Acetylthreonine	1.320
Pyridoxal	1.294
Formylmethionyl Peptide	1.290
Uridine 5′-monophosphate	1.274
Niacinamide	1.265
Glucose 6-phosphate	1.243
Xylose	1.174
Cytidine monophosphate	1.160
Histidine	1.135
Leucine	1.113
Inosine	1.096
Adenine	1.09133
Methionine	1.08293
Creatine	1.05384
Mevalonic acid	1.00851
Sarcosine	1.0041

A total of 12 enriched metabolic pathways were found in the ovaries after infections, including glyoxylate and dicarboxylate metabolism, nitrogen metabolism, and purine metabolism (Fig. 4B, Table 3).

**Table 3:**
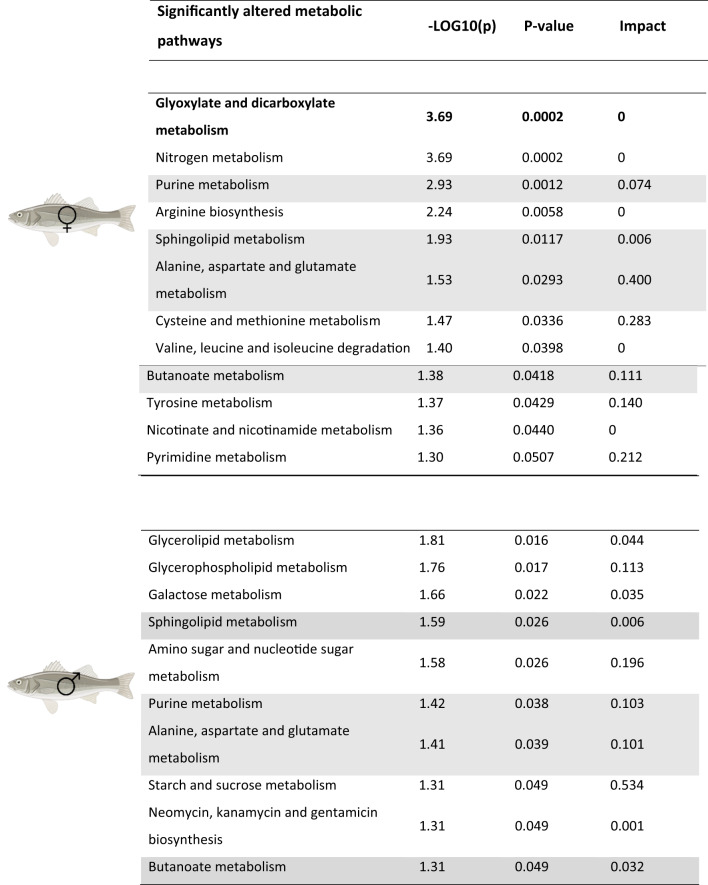
Pathway analysis results on the differentially released metabolites (DRMs) of the PLS-DA model in seabass testes and ovaries 48 h after immune stimulations. Metabolic pathways with* P*-values < 0.05 are considered significantly impacted. In grey the four common altered pathways in both sexes are highlighted

### Metabolites released in the testes after infection

In testes, 38 DRMs constituted the most variability between the infected and control groups based on VIP analysis scores higher than 1 (Table 2). Based on significance *P-*value < 0.05 and the twofold change of the volcano plot (Fig. 5A), the most abundant DRM were uridine (FC: 21.4,* P* = 0.03), GABA (FC: 13, *P* = 0.049), and n-acetyl-glucosamine 6-phosphate (FC: 9.2,* P* = 0.03) (Fig. 6; Supplementary Fig S3A). The less abundant metabolites included uric acid (FC: − 13.6,* P* = 0.009), glucose (FC: − 8.9,* P* = 0.018), N-acetylglutamine (FC: − 7.5,* P* = 0.022), glutaric acid (FC: − 6.8,* P* = 0.017), O-phosphoethanolamine (FC: − 5.2,* P* = 0.026), and glycerol 3-phosphate (FC: − 5.2, *P* = 0.016) (Fig. 6; Supplementary Fig S3B).

Pathway analysis conducted on the DRM identified 10 most significant metabolic pathways when comparing control versus infected groups, including glycerolipid metabolism, glycerophospholipid metabolism, and galactose metabolism (Fig. 5B, Table 3). Four common pathways that were altered after infection in both ovaries and testes were sphingolipid metabolism, purine metabolism, alanine, aspartate and glutamate metabolism, and butanoate metabolism.

### Transcriptome-metabolome interactions

**Fig. 7:**
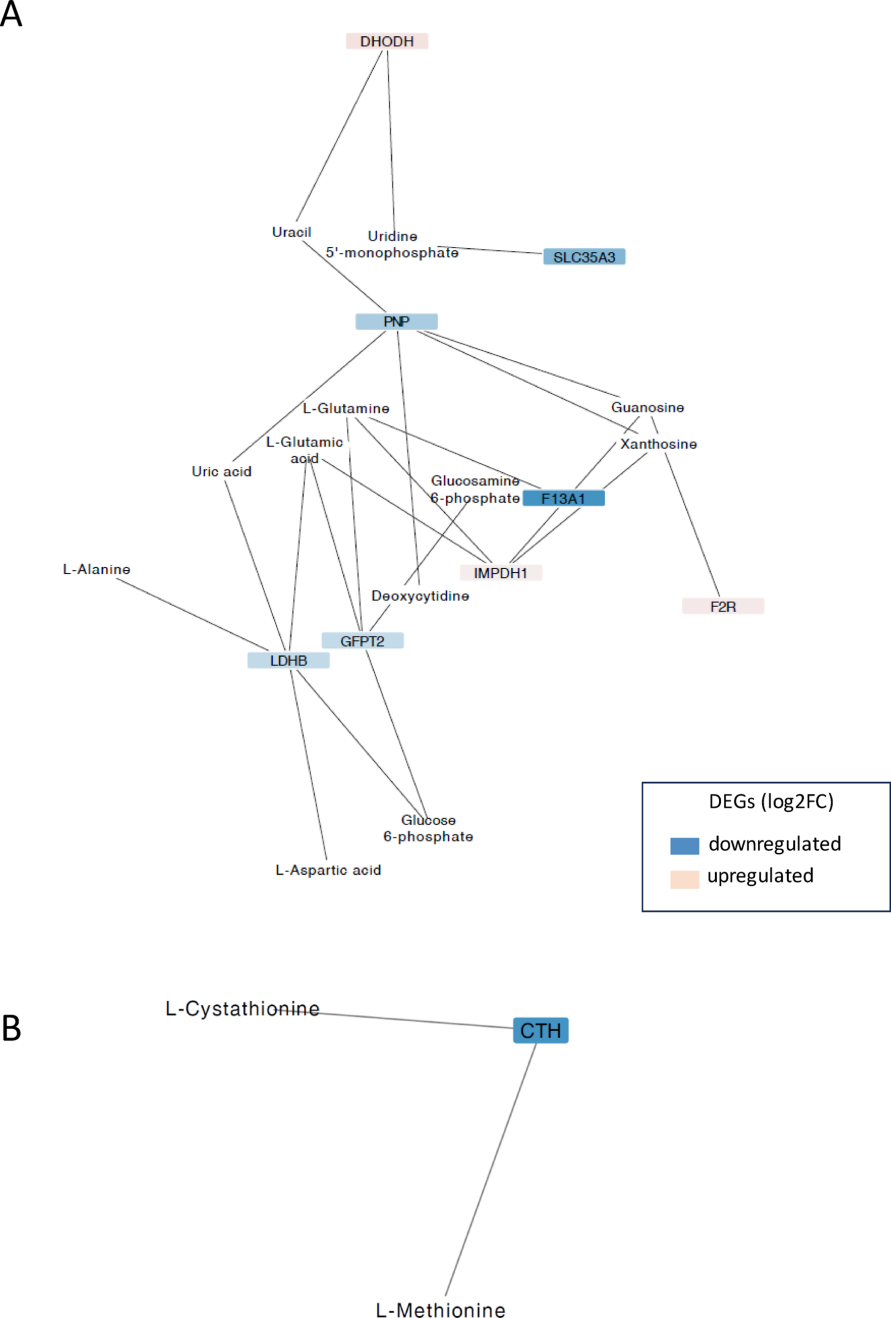
Principal network (**A**) and secondary network (**B**) generated using the Prefuse Force Directed Layout in the European sea bass ovaries 48 h of bacterial post-injection. These networks connect metabolites with VIP > 1 to differentially expressed genes (DEGs) with an adjusted *P*-value ≤ 0.05 from the comparison between infected ovaries and controls. Node colors represent gene expression changes (log2FC): Blue indicates downregulated genes, white indicates no change, and red indicates upregulated genes. In network A, log2FC values range from − 4.56 (darker blue) to 0.62 (darker red). In network B, the gene CTH shows a log2FC of − 0.55

**Fig. 8:**
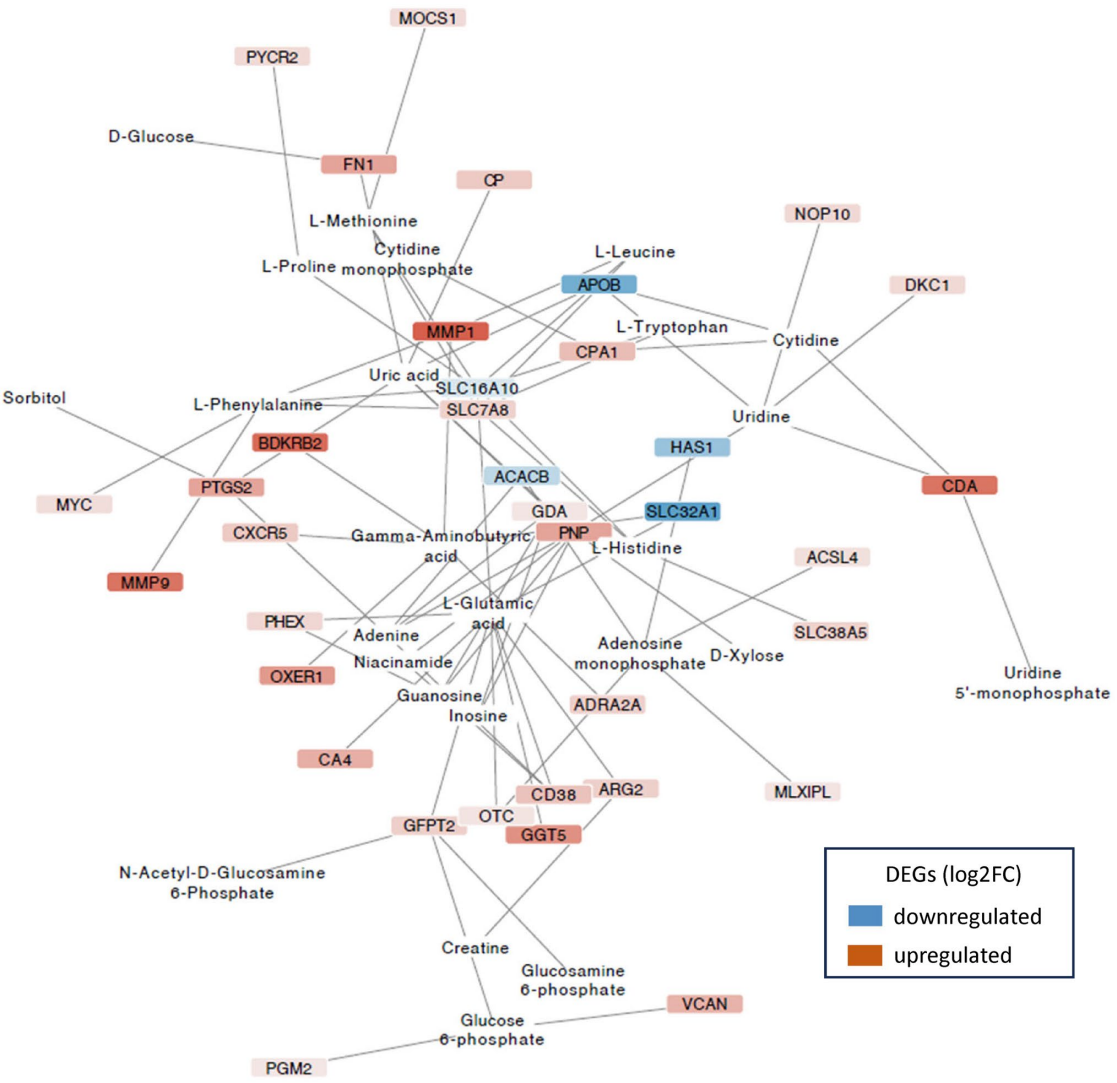
Gene-metabolite network generated using the Prefuse Force Directed Layout in the European sea bass testes 48 h of bacterial post-injection. These networks connect metabolites with VIP > 1 to differentially expressed genes (DEGs) with an adjusted *P*-value ≤ 0.001 from the comparison between infected testes and controls. Node colors represent gene expression changes (log2FC): Blue indicates downregulated genes, white indicates no change, and red indicates upregulated genes. Log2FC values range from −5.74 (darker blue) to 6.66 (darker red)

Network analysis between DEGs and the metabolites showed different results when comparing the gonadal tissues. In the case of the ovaries, the main network contains 20 nodes and 23 edges in which five DEGs were downregulated and three upregulated (Fig. 7A), whereas in the testes, the main network was more complex, containing 215 nodes and 324 edges, as having higher amount of DEGs and metabolites after the bacterial infection (Fig S4). The most stringent gene-metabolite network generated in testes (Fig. 8) showed a total of 61 nodes and 75 edges, with larger number of upregulated DEGs were found (29 *vs*. 5, respectively). These results suggested a different interaction between the transcriptome and metabolome when comparing both tissues.

In ovaries, the higher abundance of uric acid after the infection was correlated with the downregulation of two E. sea bass metabolic genes (Fig. 7A, B): Purine nucleoside phosphorylase 5a (*pnp5a*, PNP human ortholog, log2FC: − 1.96) and lactate dehydrogenase b (*ldhb*, LDHB human ortholog, log2FC: − 1.35). Furthermore, the *ldhb* gene had connections with the lower levels of both L-alanine and L-aspartic acid metabolites previously described. In addition, the lower levels of L-glutamine was connected with the downregulation of *glutamine-fructose-6-phosphate transaminase* (*gfpt2*, GFPT2 in humans, log2FC: − 1.32) and the human ortholog Coagulation Factor XIII A Chain (F13A1, log2FC: − 4.56), and the upregulation of the human ortholog Inosine Monophosphate Dehydrogenase 1 (IMPDH1, log2FC: 0.30). Finally, the lowest expression of methionine was related to the downregulation of *cystathionine gamma-lyase* (*cthl*, CTH human ortholog, log2FC: − 0.55) in a secondary network (Fig. 7B).

In the testes, the correlation network analysis (Fig. 8) the major abundance of uridine was related to the upregulation of *nop10* *ribonucleoprotein* (*nop10*, NOP10 in humans, log2FC: 1.32), Dyskerin Pseudouridine Synthase 1 (DKC1, log2FC: 1.29), *cytidine deaminase b* (*cdab*, CDA, log2FC: 5.79), Purine Nucleoside Phosphorylase (PNP, log2FC: 3.65) and with the sub-expression of Apolipoprotein B (APOB, log2FC: − 4.89). Furthermore, the higher abundance of n-acetyl-d-glucosamine 6-phosphate was connected to the upregulation of *glutamine-fructose-6-phosphate transaminase 2* (*gfpt2*, GFPT2, log2FC: 1.78). On the other hand, the lower abundance of uric acid was related in the gene-metabolite network with the upregulation of five genes, including Fibronectin 1 (FN1, log2FC: 3.65), Ceruloplasmin (CP, log2FC: 2.01), PNP (log2FC: 3.65), *guanine deaminase* (*gda*, GDA, log2FC: 0.78), *prostaglandin-endoperoxide synthase 2b* (*ptgs2b*, PTGS2, log2FC: 3.60) and the sub-expression of APOB.

## Discussion

Exposure to a high microbial burden leads to an immune response that affects homeostatic pathways involved in metabolic regulation, nutrient partitioning, behavior, thermoregulation, and hypothalamic-pituitary-adrenocortical (HPA) activity (Colditz, 2002; DePeaux & Delgoffe, 2021). The crosstalk between metabolic and immune systems has recently gained wide interest, with a focus on certain cytokines, hormones, neuropeptides, immune-related proteins, transcription factors, and glucose metabolism (DePeaux & Delgoffe, 2021; Kapnick et al., 2023; Matarese & La Cava, 2004). Nevertheless, more information is needed on the metabolic processes after the immune challenge, especially in fish, and the identification of metabolic biomarkers in the gonads. Previous studies have mainly focused on the metabolic and transcriptomic processes during gonadal development, for example, in dentex (*Dentex dentex*), Chinese sturgeon (*Acipenser sinensis*), and in Japanese grenadier anchovy (*Coilia nasus*) (Chatzifotis et al., 2004; Leng et al., 2019; Xu et al., 2016). In zebrafish, the metabolomic and transcriptomic profiles after LPS-immune stimulation were studied only in the male gonadal tissue (van Gelderen et al., 2024). In a recent study in E. sea bass indicated that following infection, testes exhibited more pronounced alterations in both the miRNome and transcriptome (van Gelderen et al., 2025). In this context, our study showed, for the first time, changes in the metabolic profile, its relation with the transcriptome, and metabolomic pathways altered in the ovaries and testes in a commercially important cultured fish species after bacterial infections, *V. anguillarum*, which is a causative agent of vibriosis responsible for severe economic losses worldwide affecting various marine and fresh/brackish water fish, bivalves and crustaceans (Frans et al., 2011; Noorian et al., 2023).

Here, we demonstrated that intraperitoneal bacterial infection was able to significantly alter metabolites and metabolic-related pathways both in the ovaries and testes. Eight metabolites were altered in the ovaries and nine in the testes. The ovaries and the testes were not releasing the same metabolites in response to infection and were showing different gene-metabolite networks. Therefore, the metabolites in the present study could potentially be used as biomarkers for each sex, especially O-phosphoethanolamine and uric acid which were inversely correlated. In particular, O-phosphoethanolamine was abundantly released in the infected ovaries, more than 750 times that of control ovaries, while it was decreased five times in the testis, compared to their respective control groups. O-phosphoethanolamine is a precursor for phosphatidylethanolamine, which is the most abundant lipid on the cytoplasmic layer of cellular membranes, playing a significant role in the immune system, involving a variety of cellular processes such as membrane fusion, cell cycle, autophagy, and apoptosis (Pavlovic & Bakovic, 2013). In comparison, O-phosphoethanolamine increased in the spleen after *Orientia tsutsugamushi* infection in mice, probably associated with recognizing the pathogen in the immune cells (Jung et al., 2015). In humans, O-phosphoethanolamine increased in the microbiota of hepatocellular carcinoma (Xue et al., 2022) and bladder cancer patients (Chen et al., 2019), revealing O-phosphoethanolamine as a potential clinical marker.

The uric acid pattern was similar to that observed for O-phosphoethanolamine. In fish, purine degradation ends with the oxidation of uric acid to allantoin by the enzyme urate oxidase, considered an important antioxidant with a potent ability as a scavenger of oxygen singlet and hydroxyl radicals (Ladisa et al., 2021). Here we observed an increase of allantoin in the ovary, but not in the testes, after the immune challenge. Similarly, zebrafish exposed to glyphosate showed an increase in uric acid in females but not in males in the liver, linked to increased expression of inflammatory-related genes in females and decreased expression of oxidative stress-related genes in males (Giommi et al., 2022). Masculinization in zebrafish induced by the progestin dydrogesterone resulted in a decrease of uric acid together with the increase of other metabolites, such as uracil. In the present study, the more abundant release of the same metabolites in testes might be important in induced male-skewed development (Jiang et al., 2019).

The uric acid metabolite also showed a different correlation with the DEGs when comparing both tissues, suggesting that it was not only an inversely correlated metabolite, but it was also differently regulated by gene expression between ovaries and testes. The higher abundance in ovaries was associated with the downregulation of *pnp5a* and *ldhb* enzymes involved in purine nucleoside degradation and pyruvate-lactate interconversion. In an immune approach, *pnp5a* was involved in innate immunity (Podok et al., 2014), and the decrease in the expression of LDHB in humans was related to major tumor progression in various cancers (Luo et al., 2024). In contrast, in testes, the lower abundance of uric acid was associated with the upregulation of five genes, including CP, *gda*, FN1, PNP, and *ptgs2b* and the downregulation of APOB gene. CP and *gda* were involved in the oxidation of Fe(II)-transferrin to Fe(III)-transferrin and the guanine metabolic process, respectively. In addition, FN1 gene was a key protein in immune response, cell adhesion, migration, and tissue repair (Ffrench-Constant et al., 1989; Han & Roman, 2006; Spada et al., 2021; Yamada & Olden, 1978). FN1 was proposed as a biomarker for various cancers, and its overexpression was linked to a poor prognostic and reduced patient survival (Pan et al., 2024; Wang et al., 2022). PNP was involved in lymphocyte purine metabolism, and mutations in this gene was related to a deficiency in the immunity of lymphocytes T and B, being linked to some autoimmune diseases, including systemic lupus erythematosus (Ghodke-Puranik et al., 2017). *Ptgs2b* was related to the production of prostaglandins (Kamal et al., 2023) that have both pro- and anti-inflammatory effects on immune cells. In zebrafish, the upregulation of this gene was observed in a rapid immune response (Sepahi et al., 2019). Finally, APOB was associated with both innate and adaptive immunity, playing a role in host defense (Sigel et al., 2012) and modulating T cells in chronic inflammatory conditions such as atherosclerosis (Nettersheim et al., 2023).

Bacterial infection increased the levels of uridine, GABA, and N-acetyl-glucosamine 6-phosphate in testes. Uridine that was linked to the upregulation of four genes and downregulation of one, has remarkable functions in tissues under stress or pathological situations (Pizzorno et al., 2002), although, in virus infection in carp, uridine was decreased in white blood cells (Tang et al., 2019). GABA enhances antimicrobial responses against intracellular bacterial infection, and its role in the immune system has been demonstrated in some fish species such as zebrafish (Zhao et al., 2016), and Olive flounder, *Paralichthys olivaceus* (Farris et al., 2022; Kim et al., 2018; Zhao et al., 2016). Consistent with the present observation, an increase in extracellular GABA, glutamate, and aspartate reflected the degree of tissue damage after inducing brain infection with *Staphylococcus aureus* (Hassel et al., 2014). Thus, the observed abundance of GABA in the testes might be a characteristic biomarker of infection. N-acetyl-glucosamine 6-phosphate appeared linked with the upregulation of *gfpt2* that played a role in the biosynthesis of UDP-N-acetylglucosamine and in the metabolic processing of fructose 6-phosphate. To date, scarce data is available on N-acetyl-glucosamine 6-phosphate and less in fish infections. In humans, it is known that a deficit of the enzyme responsible for the conversion of N-acetyl-glucosamine 6-phosphate to N-acetylglucosamine-l-phosphate leads to immunodeficiency (Lundin et al., 2015; Stray-Pedersen et al., 2014).

Gonads have immune advantages that prevent immune responses against meiotic germ cells, which is useful as a mechanism against infertility (Maddocks & Setchell, 1990). However, this does not mean that they are free of infection, which can have serious implications for the broodstock and its future offspring, especially through the procedures used by pathogens to spread via vertical transmission (Valero et al., 2018). We found common strategies for enhancing metabolomic pathways in both sexes. The most significant metabolic pathways were the metabolism of sphingolipid, butanoate, purine, alanine, aspartate, and glutamate. In many cases, the attachment and uptake of pathogenic bacteria and their development and survival within the host cells depend on sphingolipids, but at the same time, they can act as antimicrobials, inhibiting bacterial growth and formation of biofilms (Kunz & Kozjak-Pavlovic, 2019). Synthesis and metabolism of short-chain fatty acids by the microbiome modulate inflammatory cytokine activity, especially by increased butanoate and propionate production (Karpe et al., 2021).

Males and females exhibit different gonadal physiology, different profiles of sex hormones and variations in immune regulation (Caballero-Huertas et al., 2024; Valero et al., 2018). Thus, a better understanding of the molecular mechanisms of immunity can help to develop screening methods to cope with infections typically found in aquaculture (van Gelderen et al., 2024). However, it is essential to distinguish the molecular processes that occur in each sex. In most species, the prevalence and intensity of infections are higher in males than females (Klein, 2000; Valero et al., 2018). In this regard, van Gelderen et al. (2025) reported that in European sea bass, testis exhibits altered miRNAome and transcriptome after bacterial infection compared to the ovary. In particularly, males showed approximately 26% more differentially expressed genes in testicular genes compared to females while miRNAs were only altered in testes. This major alteration in the testes was reflected in our study, with the major complexity of the gene-metabolite network obtained in the testes. However, due to the great diversity of fish taxa, their immune mechanisms, as well as their life traits concerning diverse reproductive strategies, fertilization modes, breeding, and parental behaviors, the ability to fight infections can be varied and complex (Caballero-Huertas et al., 2024; Wootton & Smith, 2014). As an example of the present induced infection experiment, in European sea bass, *Vibrio gigantis* growth was seen in the female gonads of deceased fish, whereas no bacterial growth was found in the male gonads (Yilmaz et al., 2023). In the present data, the infection in ovaries primarily activated metabolic pathways related to glyoxylate and dicarboxylate metabolism, nitrogen metabolism, and purine metabolism. In contrast, the infection in the testes mainly affected glycerolipid metabolism, glycerophospholipid metabolism, and galactose metabolism. Our data corroborated the existence of sexual dimorphism in the metabolite released in the fish gonads. Similar evidence was already observed in other tissues. For example, livers of migrating sockeye salmon (*Oncorhynchus nerka*) display sex-linked endocrine perturbation (Benskin et al., 2014). Furthermore, there is evidence for a strong sexual dimorphism in medaka involving cellular and molecular processes of hepatocytes, including protein synthesis, amino acid, lipid, and polysaccharide metabolism, along with steroidogenesis and detoxification after hepatotoxic exposures (Qiao et al., 2016). In largemouth bass (*Micropterus salmoides*), sexual differences were observed in the liver with respect to different biochemical responses (Li et al., 2024). Thus, confirming the importance of sex as a relevant factor that interacts with the immune system in fish.

## Conclusions

The present study of infected European seabass gonads identified potential metabolites as biomarkers for females (i.e., uric acid, O-phosphoethanolamine, allantoin, and acetoacetic acid) and males (i.e., uridine, N-acetylglucosamine-6-Phosphate, GABA). Thus, sexual dimorphic responses in the metabolites released due to infections offer new perspectives for developing sex-specific immune therapies and resistant phenotypes in aquaculture. Given sex differences in fish immune responses, improving infection protocols could help reduce the impact of bacterial diseases on the aquaculture industry and promote more sustainable systems.

## Supplementary Information

Below is the link to the electronic supplementary material.

Supplementary file1 (PPTX 1235 KB)

## Data Availability

No datasets were generated or analysed during the current study.
