# Supplementary figures and images for: Identifying sex-linked metabolomic biomarkers in fish gonads after bacterial infection
Source: Metabolomics. 2025 Nov 15;21(6):167. doi: 10.1007/s11306-025-02356-7 (PMC12619773; doi:10.1007/s11306-025-02356-7)

## Slide 1
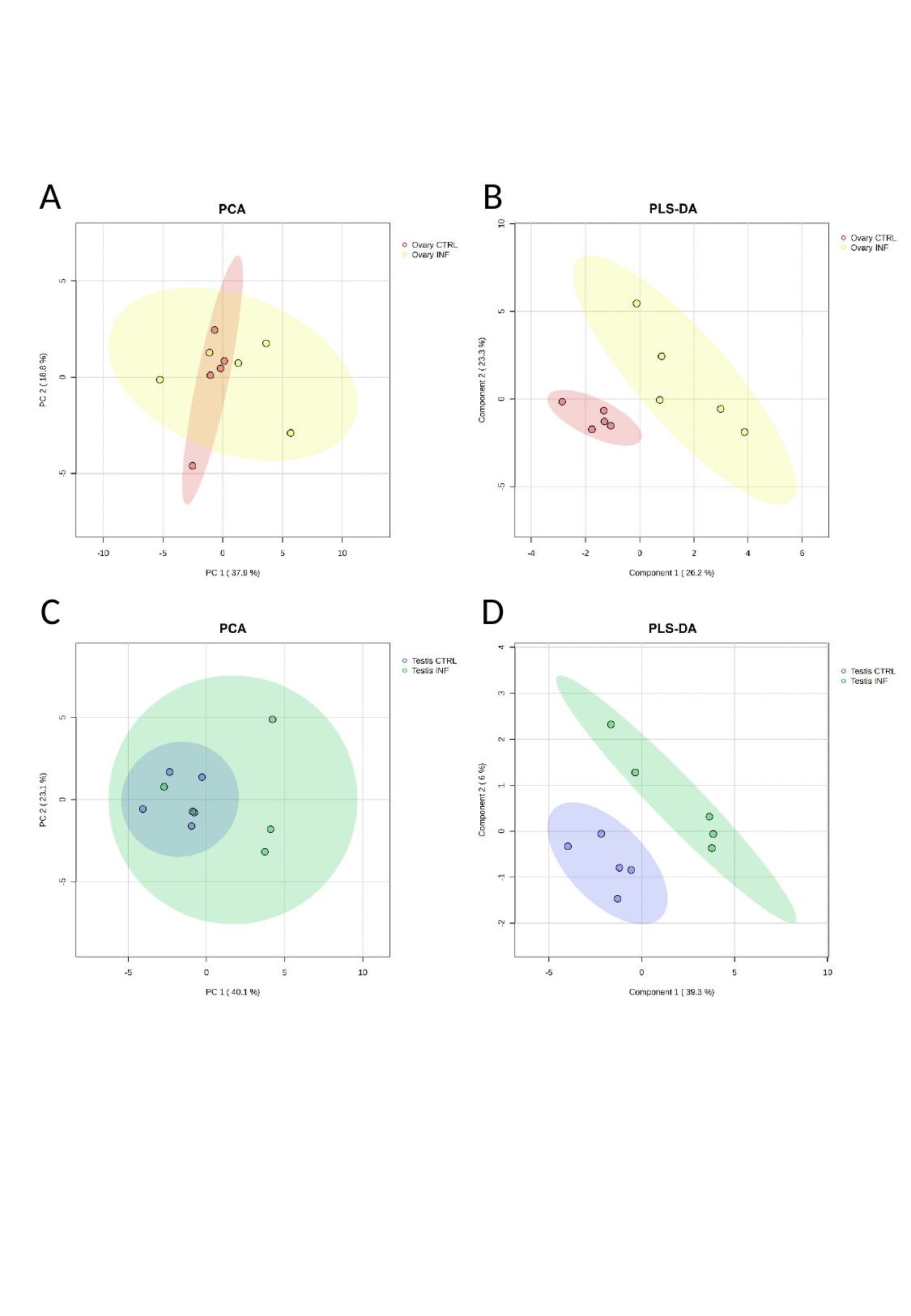

A
B
C
D

## Slide 2
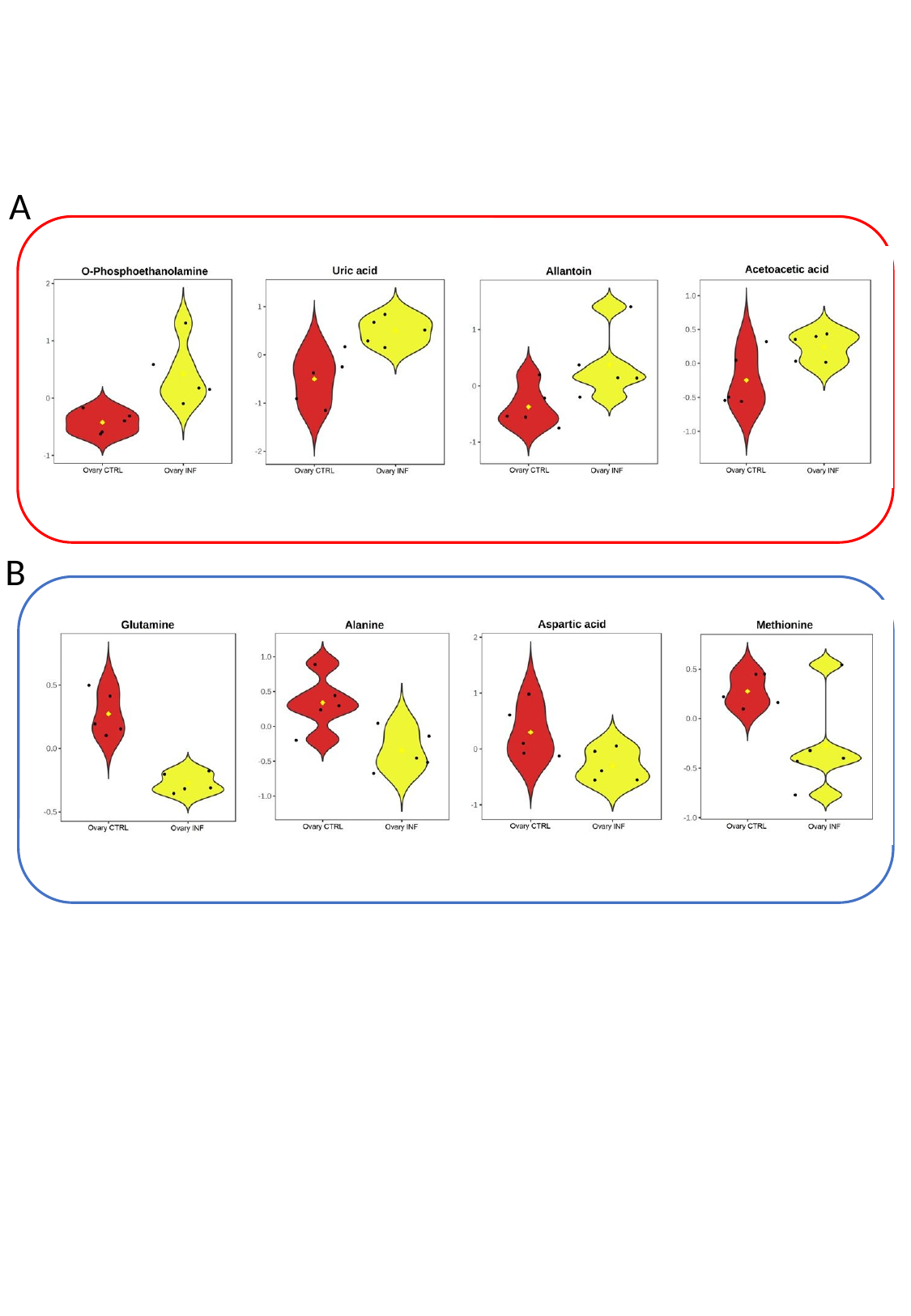

A
B

## Slide 3
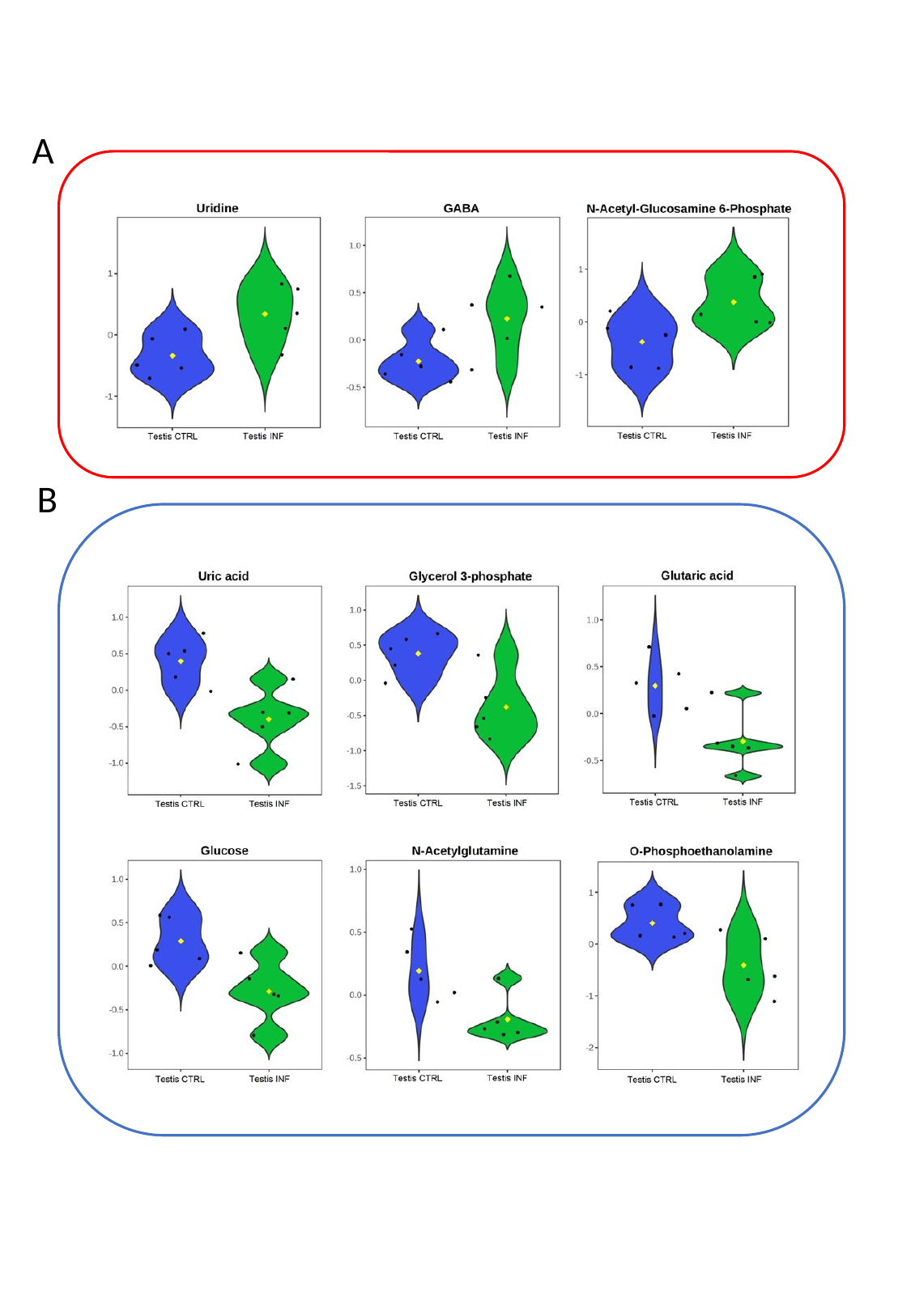

A
B

## Slide 4
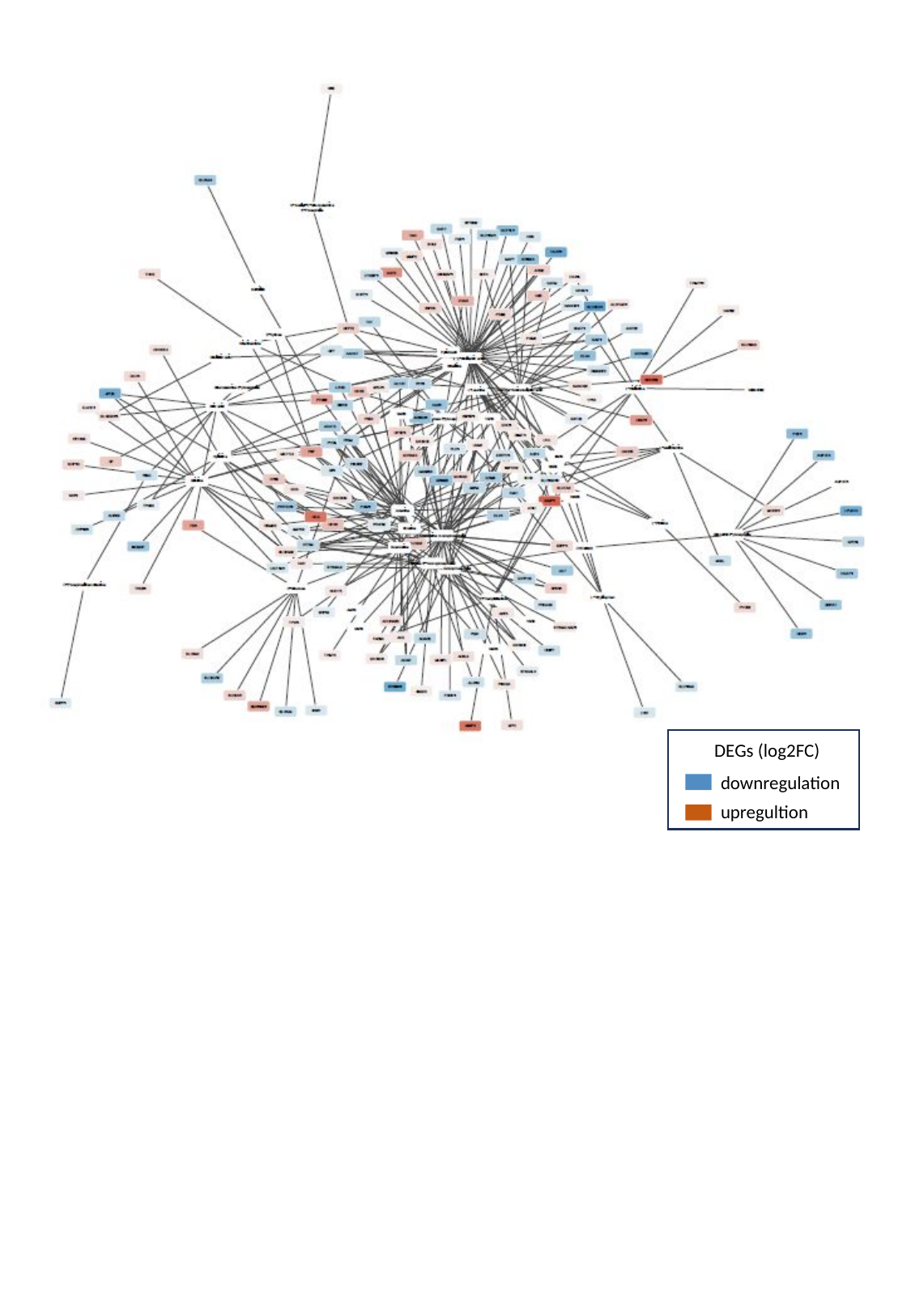

DEGs (log2FC)
downregulation
upregultion

Supplement: Supplementary file 1 — Supplementary file1 (PPTX 1235 KB) [file 11306_2025_2356_MOESM1_ESM.pptx]
